# A Disposable Amperometric Sensor Based on High-Performance PEDOT:PSS/Ionic Liquid Nanocomposite Thin Film-Modified Screen-Printed Electrode for the Analysis of Catechol in Natural Water Samples

**DOI:** 10.3390/s17081716

**Published:** 2017-07-26

**Authors:** Francis D. Krampa, Yaw Aniweh, Gordon A. Awandare, Prosper Kanyong

**Affiliations:** 1 West African Centre for Cell Biology of Infectious Pathogens (WACCBIP), University of Ghana, Legon, Accra, Ghana; fkrampa@gmail.com (F.D.K.); aniweh@gmail.com (Y.A.); gawandare@ug.edu.gh (G.A.A.); 2 Department of Biochemistry, Cell & Molecular Biology, University of Ghana, Legon, Accra, Ghana; 3 Nanotechnology & Integrated Bioengineering Centre, Ulster University, Jordanstown BT37 0QB, UK

**Keywords:** room temperature ionic liquids, PEDOT:PSS, disposable sensors, cyclic voltammetry, electrochemical impedance spectroscopy, screen-printed electrodes, conducting polymers, nanocomposites, hexacyanoferrate, sessile contact angle measurement

## Abstract

A conducting polymer-based composite material of poly(3,4-ethylenedioxythiophene) (PEDOT): poly(4-styrenesulfonate) (PSS) doped with different percentages of a room temperature ionic liquid (IL), 1-ethyl-3-methylimidazolium tetrafluoroborate ([EMIM][BF_4_]), was prepared and a very small amount of the composite (2.0 µL) was drop-coated on the working area of a screen-printed carbon electrode (SPCE). The SPCE, modified with PEDOT:PSS/IL composite thin-film, was characterized by cyclic voltammetry (CV), electrochemical impedance spectroscopy (EIS), scanning electron microscopy (SEM), profilometry and sessile contact angle measurements. The prepared PEDOT:PSS/IL composite thin-film exhibited a nano-porous microstructure and was found to be highly stable and conductive with enhanced electrocatalytic properties towards catechol, a priority pollutant. The linear working range for catechol was found to be 0.1 µM–330.0 µM with a sensitivity of 18.2 mA·mM·cm^−2^ and a calculated limit of detection (based on 3× the baseline noise) of 23.7 µM. When the PEDOT:PSS/IL/SPCE sensor was used in conjunction with amperometry in stirred solution for the analysis of natural water samples, the precision values obtained on spiked samples (20.0 µM catechol added) (*n* = 3) were 0.18% and 0.32%, respectively, with recovery values that were well over 99.0%.

## 1. Introduction

Over the past 20 years, the development of sensitive and real-time analysis of phenolic compounds has received substantial scientific interest due to their high toxicity on the ecosystem, environment as well as human health [1]. Besides this, phenolic compounds as highly toxic organics has been extensively utilized in various industrial products including flavors, pharmaceuticals, antioxidants, agrochemicals, and polymerization inhibitors [1,2,3,4]. Among phenolic compounds, catechol, which is an ortho isomer of benzenediols, has been listed as a priority pollutant by both the European Union and the US Environmental Protection Agency [5,6] because it has a poor biodegradability and is extremely toxic to human health and the ecosystem [7,8]. Therefore, there is the need for the development of analytical tools that allow for simple, rapid, and real-time analysis of trace levels of catechol in environmental samples.

Currently, various analytical methods including mass spectrometry, gas and high-performance liquid chromatography, electrochemiluminescence, fluorescence, and electrochemical methods [9,10,11,12] have been used to analyze catechol. Even though these methods are sensitive towards catechol, they are usually not only time-consuming, laborious, and require skilled-personnel to operate, but also involve complicated operational procedures that makes them unsuitable for point-of-need applications. Owing to the electroactive nature of catechol, the use of electrochemical techniques, especially at modified electrodes, are most attractive because they give rapid response and are simple, relatively inexpensive, selective, and sensitive [12,13,14,15]. Different nanomaterials including carbon nanomaterials, nanoparticles, metals and metal oxides, conducting polymers [16,17,18,19] including electrode pre-treatments and/or modifications have been developed for the quantification of catechol [19,20].

The use of conducive polymers in sensor design provides numerous advantages because these materials are relatively inexpensive and environmentally friendly, exhibit good charge storage capacity, and biocompatibility for biomolecules immobilization, wide potential windows and excellent electrical conductivity particularly when doped [21]. Among the different types of conductive polymers, the polythiophene-derived macromolecule species poly(3,4-ethylenedioxythiophene) (PEDOT) is known to be the best in terms of conductivity, stability, and processability [21,22]. Also, colloidal dispersions of PEDOT can be readily made through the addition of poly(4-styrenesulfonate) (PSS) to form the doped compound PEDOT:PSS. This doped version of the polymer has excellent conductivity and exhibits good mechanical properties [23]; thus, it has been applied to the development of various devices and sensors [24,25,26].

Room temperature ionic liquids (ILs) are organic/inorganic salts that are liquid at room temperature and are usually considered to be ‘green solvents’. They are known to have good chemical stability, high ionic conductivity, negligible vapor pressure, low flammability and have been used in many technological fields [14]. Because of the high affinity of ILs with conductive polymers and their ability of supramolecular ordering, we envisaged the use of ILs as dopants in the conductive polymer PEDOT:PSS to enhance the charge transfer rate of PEDOT:PSS for catechol. Consequently, in this study, we prepared different percentages of the ionic liquid, 1-ethyl-3-methylimidazolium tetrafluoroborate ([EMIM][BF_4_]) in PEDOT:PSS. Thin films of PEDOT:PSS/ionic liquid were prepared by casting the composite on the working area of a screen-printed carbon electrode (SPCE) and dried at 40 °C for about 1 h. Overall, the specific advantages of screen-printed sensors such as miniaturization, disposability, and low-cost, and the synergistic effect of PEDOT:PSS and [EMIM][BF_4_] are assembled to fabricate a low-cost, disposable, and simple sensor. The applicability of the sensor as a useful analytical tool was demonstrated through analysis of catechol in natural water samples. Details of the sensor fabrication, assembly, and characterization are described and discussed.

## 2. Experimental

### 2.1. Apparatus and Reagents

**Scheme 1:**
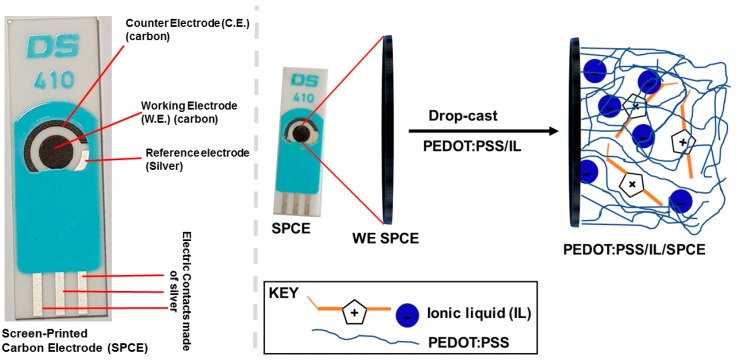
Schematic representation of the Screen-Printed Carbon Electrode (SPCE) (**left**) and procedure for fabricating the PEDOT:PSS/20%IL/SPCE sensor (**right**).

Electrochemical experiments were conducted using PGSTAT204 Autolab Potentiostat/Galvanostat/EIS FRA32M Module (Metrohm-Autolab, The Netherlands) with Nova 2.1 Software for data acquisition and experimental control. Electrochemical impedance spectroscopy in 5.0 mM potassium hexacyanoferrate ([Fe(CN)_6_]^3−^/[Fe(CN)_6_]^4−^) was carried out at open circuit within the frequency range of 100 kHz–0.1 Hz at an applied potential of 0.25 V. The disposable screen-printed carbon electrodes (Ref DS 410) utilized in the sensor design have a carbon working electrode, carbon counter electrode, and silver reference electrode (Scheme 1) and were purchased from DropSens, Asturias, Spain. Scanning electron microscopy (SEM) was performed by JEOL JSM-610PLUS/~LA SEM (JEOL Ltd., Tokyo, Japan). Further surface analysis was performed using Bruker DektakXT^®^ Stylus profilometer (Bruker Optics, Ettlingen, Germany). Sessile contact angle measurements were performed using CAM200 Optical Contact Angle Meter (KSV Instruments Ltd., Helsinki, Finland).

Poly(3,4-ethylenedioxythiophene) (PEDOT): poly(4-styrenesulfonate) (PSS), 1-ethyl-3-methylimidazolium tetrafluoroborate ([EMIM][BF_4_]), hexacyanoferrate ([Fe(CN)_6_]^3−^/[Fe(CN)_6_]^4−^), phosphate buffered saline (PBS) tablets, and catechol were purchased from Sigma-Aldrich, St. Louis, MO, USA. All other chemicals were of analytical grade and used without further purification.

### 2.2. Procedures

#### 2.2.1. Fabrication of PEDOT:PSS/20%IL/SPCE

The bare Screen-Printed Carbon Electrode (SPCE) was modified by drop-coating 2.0 μL each of PEDOT:PSS and ionic liquid (IL) ([EMIM][BF_4_]), and dried at 40 °C for 1 h to form PEDOT:PSS/SPCE and IL/SPCE, respectively. Different percentages of IL (v/v) (1.0, 2.0, 5.0, 10.0, 20.0, 30.0, and 50.0%) in PEDOT:PSS were also prepared and 2.0 μL of the composite drop-coated on the SPCE to form PEDOT:PSS/IL/SPCE and were allowed to dry as previously described. The fabrication process is illustrated in Scheme 1. The surfaces of all the modified SPCEs were thoroughly rinsed in PBS to remove any unbound species. Once prepared, the sensors were stored in room temperature conditions.

#### 2.2.2. Sessile Contact Angle Measurement

The contact angle measurements were carried out by the sessile drop technique; a water droplet was placed onto a flat surface of the bare SPCE and PEDOT:PSS/IL composite modified SPCE, and the contact angle of the droplet with the surface measured. Reported values are the average contact angle (right and left) of 10 droplets. During the measurement time (~50 s), no change in contact angle was observed. A variation of 5° is generally considered to be sufficient to differentiate materials [13].

## 3. Results and Discussion

### 3.1. Optimisation of the Percentage of IL in PEDOT:PSS/IL Composite

**Figure 1:**
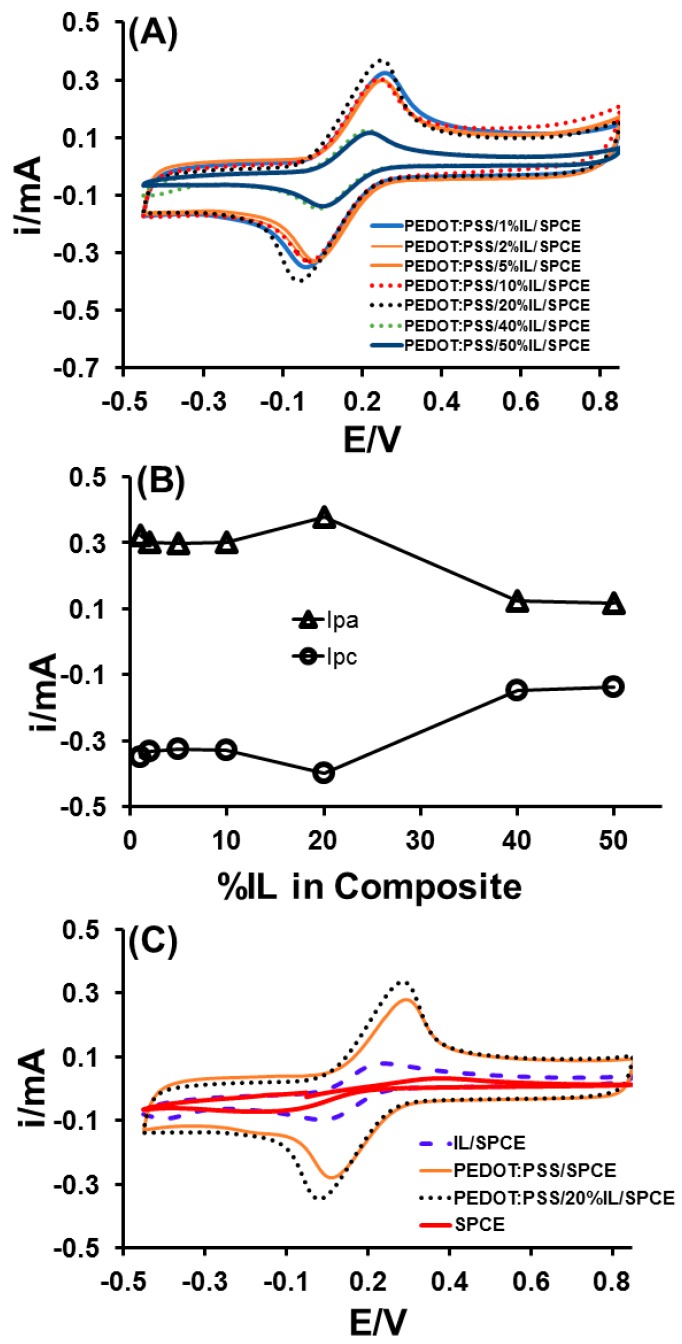
(**A**) Cyclic voltammograms of PEDOT:PSS/IL/SPCE prepared with different amounts of ionic liquid (IL) (1.0, 2.0, 5.0, 10.0, 20.0, 40.0, and 50%) in PEDOT:PSS/IL composite; (**B**) Plot of anodic (*I_pa_*) and cathodic (*I_pc_*) peak currents for [Fe(CN)_6_]^3−^/[Fe(CN)_6_]^4−^ vs. amount of IL (%) in PEDOT:PSS/IL composite; (**C**) cyclic voltammetries (CVs) of SPCE, IL/SPCE, PEDOT:PSS/SPCE and PEDOT:PSS/20%IL/SPCE. All CVs were recorded in 5.0 mM hexacyanoferrate ([Fe(CN)_6_]^3−^/[Fe(CN)_6_]^4−^) in phosphate buffered saline (PBS) (pH 7.4) containing 0.1 M KCl.

To ascertain the amount of IL in PEDOT:PSS required for optimum electrocatalytic response of the modified SPCE, composites with different percentages of IL (1.0, 2.0, 5.0, 10.0, 20.0. 40.0, and 50.0%) in PEDOT:PSS were formulated and used to fabricate PEDOT:PSS/IL/SPCE sensors. Thereafter, the voltammetric responses from the PEDOT:PSS/IL/SPCE sensors were measured in PBS (pH 7.4) containing 5.0 mM hexacyanoferrate ([Fe(CN)_6_]^3−^/[Fe(CN)_6_]^4−^) and 0.1 M KCl. It should be mentioned that a similar procedure was used to evaluate the PEDOT:PSS/SPCE and IL/SPCE sensors. Figure 1A shows cyclic voltammograms recorded at SPCEs modified with different percentages of IL in PEDOT:PSS while Figure 1B shows a plot of the peak currents vs. the percentage of IL in the composites formulated. It can be seen in Figure 1A,B that the voltammetric peaks increased gradually from 1.0% IL up to 20.0% IL. Subsequent increases in the percentage of IL did not show any increase in the voltammetric response of the modified sensors. Consequently, 20.0% IL was chosen as the optimum amount of IL required to be present in PEDOT:PSS/IL composite to give the highest electrocatalytic response. Figure 1C shows a comparison of voltammograms recorded using the bare SPCE, IL/SPCE, PEDOT:PSS/SPCE, and PEDOT:PSS/20%IL/SPCE sensors in 5.0 mM ([Fe(CN)_6_]^3−^/[Fe(CN)_6_]^4−^) solution. In comparison to both PEDOT:PSS/SPCE and IL/SPCE sensors, the anodic peak current (*I_pa_*) and cathodic peak current (*I_pc_*) of the PEDOT:PSS/20%IL/SPCE sensor was more enhanced with well-defined voltammetric peaks; this enhancement in electrocatalytic properties is attributed to the synergistic effect of the PEDOT:PSS and IL. Consequently, the PEDOT:PSS/20%IL/SPCE sensor was used for further studies.

### 3.2. Characterisation of SPCE and PEDOT:PSS/20%IL/SPCE Sensor

#### 3.2.1. Cyclic Voltammetry

**Figure 2:**
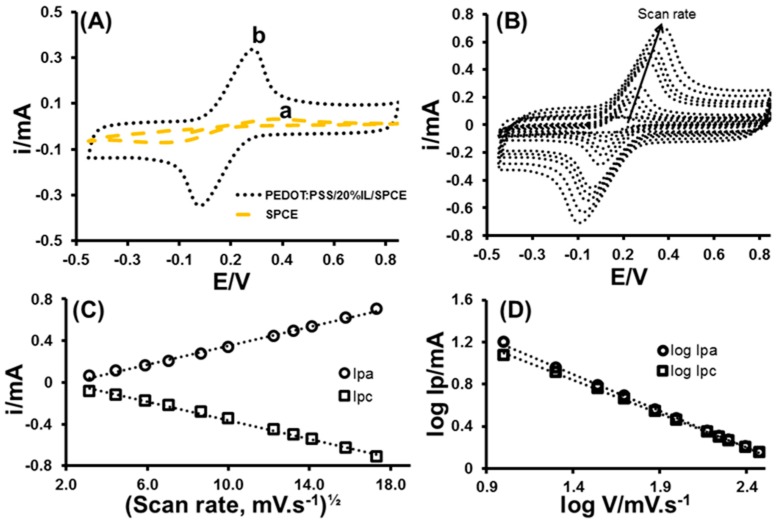
(**A**) Cyclic voltammograms recorded using SPCE (curve a) and PEDOT:PSS/20%IL/SPCE sensor (curve b) at 100 mV·s^−1^ scan rate; (**B**) CVs recorded using PEDOT:PSS/20%IL/SPCE sensor at 10, 20, 35, 50, 75, 100, 150, 175, 200, 250, and 300 mV·s^−1^ scan rates; (**C**) Peak current vs. square root of scan rate; (**D**) log I_p_ vs. log *V*. All CVs were recorded in 5.0 mM hexacyanoferrate ([Fe(CN)_6_]^3−^/[Fe(CN)^6^]^4−^) in PBS (pH 7.4) containing 0.1 M KCl.

Figure 2A shows a comparison of cyclic voltammograms recorded at the bare SPCE and PEDOT:PSS/20%IL/SPCE sensor in PBS (pH 7.4) containing 5.0 mM [Fe(CN)_6_]^3−^/[Fe(CN)_6_]^4−^ and 0.1 M KCl at a scan rate of 100 mV·s^−1^. As expected, when compared with what occurred on the bare SPCE (curve a, Figure 2A), the PEDOT:PSS/20%IL/SPCE sensor (curve b, Figure 2A) exhibited a characteristic increase of both the anodic and cathodic peak currents for [Fe(CN)_6_]^3−^/[Fe(CN)_6_]^4−^ redox couple, thus, confirming the successful modification of the SPCE with the composite. Higher peak currents and a smaller peak-to-peak potential separation (Δ*E_p_*) were observed at the PEDOT:PSS/20%IL/SPCE sensor (*I_pa_* = 336.8 µA, *I_pc_* = 345.9 µA; Δ*E_p_* = 202.6 mV) when compared with the bare SPCE (*I_pa_* = 32.4 µA, *I_pc_* = 69.9 µA; Δ*E_p_* = 346.6 mV). This is attributed to the higher electrocatalytic properties of the PEDOT:PSS/IL composite which led to an increase of the total active area of the modified electrode. The presence of the PEDOT:PSS/IL composite produced a negative shift in the anodic potential and a positive shift in the cathodic potential, giving rise to a smaller peak-to-peak separation (Δ*E_p_* = 202.6 mV). This more than ten-fold increase in the anodic peak current and five-fold increase in the cathodic peak current for [Fe(CN)_6_]^3−^/[Fe(CN)_6_]^4−^ can be attributed to the electrocatalytic effect of the PEDOT:PSS/IL composite.

The effect of scan rate (*v*) on the voltammetric behavior of the PEDOT:PSS/20%IL/SPCE sensor was also examined by CV (Figure 2B). At the scan rates investigated (10.0 to 300.0 mV·s^−1^), a plot of the square root of the scan rate (*v*) vs. the anodic (*I_pa_*) and cathodic (*I_pc_*) peak currents exhibited a linear relationship (Figure 2C), which is typical of a diffusion-controlled process [27,28,29]. A linear relationship was also observed when absolute values of both log *I_pa_* and log *I_pc_* were plotted against log *v* (Figure 2D) with slope values of 0.70 and 0.64, respectively. These slope values are comparable with the theoretically expected value of 0.5 for purely diffusion-controlled currents [27,28,29]; thus, confirming that the electrochemical process is diffusion-controlled and that the surface of the modified SPCE was not fouled.

#### 3.2.2. Electrochemical Impedance Spectroscopy

**Figure 3:**
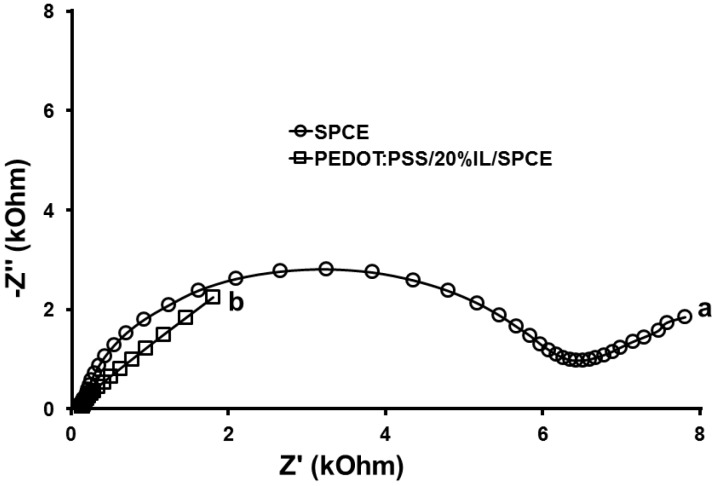
Nyquist plots observed for electrochemical impedance spectroscopy (EIS) at SPCE (curve a) and PEDOT:PSS/20%IL/SPCE sensor (curve b) in PBS (pH 7.4) containing 5.0 mM [Fe(CN)_6_]^3−^/[Fe(CN)_6_]^4−^ and 0.1 M KCl.

The interface properties of the bare SPCE and PEDOT:PSS/20%IL/SPCE sensor were further characterized by Faradaic electrochemical impedance spectroscopy (EIS) in the presence of 5.0 mM hexacyanoferrate [Fe(CN)_6_]^3−^/[Fe(CN)^6^]^4−^ (Figure 3). The impedance spectrum associated with the bare SPCE (curve a, Figure 3) consists of a semicircle part in the high frequency region and a linear part in the low frequency region, corresponding to electron transfer and diffusion processes, respectively. The diameter of the semicircle represents the charge-transfer resistance (R_CT_) at the surface of the electrode [27]. At the bare SPCE (curve a, Figure 3), a semicircle with a larger diameter was obtained. However, on the PEDOT:PSS/20%IL/SPCE sensor (curve b, Figure 3), the diameter of the semicircle was negligible. This significant change in R_CT_ value is attributed to the enhanced charge-transfer rate across the modified interface and the large surface area provided by the PEDOT:PSS/IL composite. This impedance results agree with the results obtained from the cyclic voltammetric measurements; thus, confirming the successful modification of the SPCE.

#### 3.2.3. Scanning Electron Spectroscopy and Profilometry

**Figure 4:**
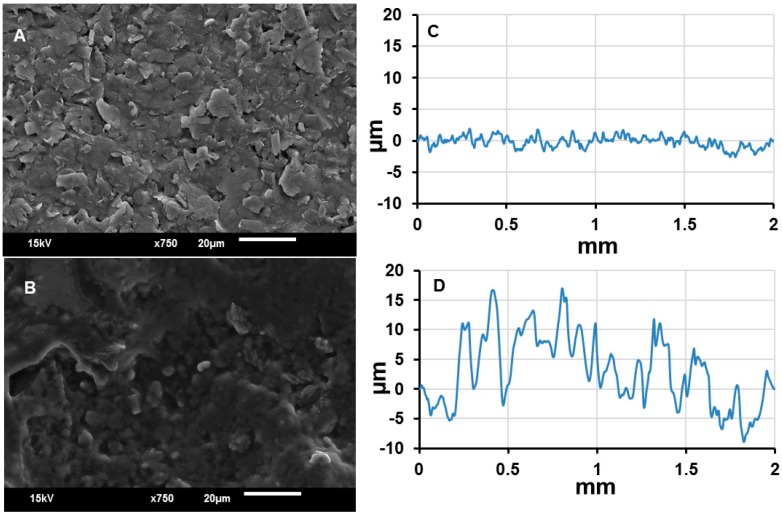
(**A**,**B**) scanning electron microscopy (SEM) images of SPCE and PEDOT:PSS/20%IL/SPCE sensor and (**C**,**D**) their corresponding surface roughness obtained from profilometry.

Additionally, the morphological features of both the bare SPCE and PEDOT:PSS/20%IL/SPCE sensor were characterized by scanning electron microscopy (SEM) as well as profilometry. Figure 4A,B show the view of the SPCE and PEDOT:PSS/20%IL/SPCE sensor, respectively. The morphology of the bare SPCE is typical for graphite materials with grains that are stacked in flakes. As shown in Figure 4B, a uniform film was formed on the electrode surface, indicating successful deposition of the PEDOT:PSS/IL composite. When compared with the bare SPCE (Figure 4A), the PEDOT:PSS/20%IL/SPCE sensor (Figure 4B) showed a highly porous morphology, consisting of several interconnected ginger-like dots; this greatly increased the surface area of the modified electrode. Profilometry measurements revealed that the average surface roughness of the SPCE (Figure 4C) and PEDOT:PSS/20%IL/SPCE sensor (Figure 4D) were 1.44 µm and 6.29 µm, respectively; these surface roughness values agree with the SEM images.

#### 3.2.4. Sessile Contact Angle Measurements

In addition to these, the measurement of the water contact angle for the PEDOT:PSS/20%IL thin film on the surface of the SPCE was performed. The contact angle of water at the surface of the bare SPCE was found to be ~74.3°. However, it decreased after coating the SPCE with PEDOT:PSS/20%IL composite to ~50.8°. This increase in the hydrophilicity of the coated electrode means that the properties of the PEDOT:PSS/20%IL composite can be manipulated in buffer solution; thus, making it a suitable surface for the immobilization of biomolecules. This is of considerable relevance for a variety of applications including sensors for biomedical applications, as well as studying biointerfaces [13].

### 3.3. Application of PEDOT:PSS/20%IL/SPCE to Catechol Analysis

#### 3.3.1. Cyclic Voltammetry

**Figure 5:**
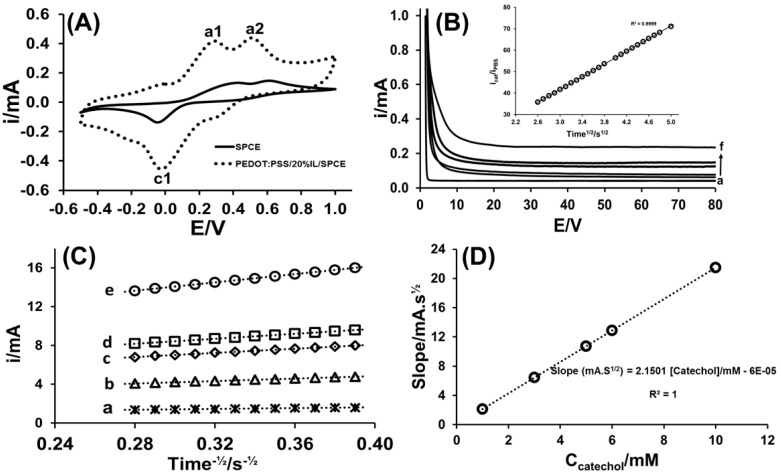
(**A**) Cyclic voltammograms recorded using the bare SPCE and PEDOT:PSS/20%IL/SPCE sensor in 5.0 mM catechol solution in PBS (pH 7.4) at a scan rate of 100 mV·s^−1^; (**B**) Chronoamperograms obtained at PEDOT:PSS/20%IL/SPCE sensor in the presence of (a) 0; (b) 1.0; (c) 3.0; (d) 5.0; (e) 6.0; and (f) 10.0 mM catechol in PBS (pH 7.4). Insert; *i_cat_*/*i_PBS_* vs. *t*^½^ plot derived from chronoamperomeric data for PBS (a) and 1.0 mM catechol (b); (**C**) Linear segments of plot *i* vs. *t*^−½^ for (a) 1.0; (b) 2.0; (c) 5.0; (d) 6.0; and (e) 10.0 mM catechol and; (**D**) plot of the slopes from graph C vs. concentration of catechol.

Figure 5A shows cyclic voltammograms for catechol at the bare SPCE and PEDOT:PSS/20%IL/SPCE sensor, respectively. During the forward scan, two prominent oxidation peaks at 0.27 V (a1) and 0.50 V (a2) were observed on the PEDOT:PSS/20%IL/SPCE sensor. The anodic peak (a1) can be attributed to the formation of *o*-semiquinone intermediates while the second another peak (a2) pertains to the oxidation of the catechol to *o*-quinone [30]. Previous studies identified the formation of the *o*-semiquinone and found the redox potential of catechol/*o*-semiquinone pair to be 0.53 V [30], which agrees with this finding. On the reverse scans, a cathodic peak (c1 = 0.01 V) on the PEDOT:PSS/20%IL/SPCE sensor was observed. This cathodic peak (c1) corresponds to the reduction of the *o*-quinone [31]. A peak current ratio (*I_pc_*_1_/*I_pa_*_2_) for the repetitive recycling of potential was found to be near unity, which is a criterion for the stability of *o*-quinone produced at the surface of the electrode [32,33]. These findings agree with the oxidation of catechol at similar surfaces [31,32,33]. The two oxidation peaks (a1 and a2) and one cathodic peak (c1) broadened and shifted to more positive potentials (a1 = 0.44 V, a2 = 0.63 V, c1 = 0.04 V) with a significant decrease in the peak currents at the bare SPCE. In comparison to what occurred at the bare SPCE, the PEDOT:PSS/20%IL/SPCE sensor exhibited a characteristic increase of both the anodic and cathodic peak currents for catechol. The enhanced prominence and the shifts in peak potentials to less positive values, and the more than four-fold increase in peak currents are attributed to the electrocatalytic properties of the PEDOT:PSS/20%IL composite.

The effect of scan rate on the voltammetric behavior of catechol at the PEDOT:PSS/20%IL/SPCE sensor was examined by CV and the two oxidation peaks and one reduction peak currents increased linearly with increasing scan rate; thus, suggesting a behavior consistent with surface confined voltammetry and corresponding ‘thin-layer’ type voltammetry [13].

To further evaluate the electrochemical behavior of the PEDOT:PSS/20%IL/SPCE sensor, the influence of scan rate on both the anodic peak potentials and cathodic peak potential of catechol were analyzed. With an increase in scan rate, the anodic peak potential shifted towards a positive value and a linear relationship was observed in the range of 10 to 300 mV·s^−1^. The equation of this behavior for *E_pa_*_2_ can be expressed as:

(1) $$E_{pa2}\left(V\right)=0.183\log v\left(V.s^{-1}\right)+0.709;\;R^{2}=0.9995.$$

According to Laviron’s expression for an electrochemical process [34,35], *E_p_* is governed by:

(2) $$Ep=E^{0\prime }+\frac{\left(2.303RT\right)}{\left(\propto n\prime \;F\right)}log\frac{\left(RTk^{0}\right)}{\left(\alpha \;n\prime \;F\right)}+\;\frac{\left(2.303RT\right)}{\left(\propto n'\;F\right)}$$

where *v* is the scan rate, *n*′ is the number of electrons transferred before the rate-determining step, α is the transfer coefficient, *E*^0^′ is the formal standard redox potential, and *k*^0^ is the standard heterogeneous rate constant of the reaction, and the other symbols have their usual meaning. The value of *αn*′ can be calculated using the slope of *E_pa_*_2_ vs. log *v* plot (here slope = 0.183). Taking *R* = 8.314 J·K^−1^·mol^−1^, *T* = 298 K, and *F* = 96480 C·mol^−1^, the value of *αn*′ was calculated to be 0.32.

According to Bard and Faulkner [36],

(3) $$\alpha =\frac{\left(47.7\right)}{\left(Ep-Ep/₂\right)}\;mV$$

where *Ep* − *Ep*/2 is the potential at which the current is at half its peak value. From this, the value of α was calculated to be 0.15. Consequently, the number of electrons (*n*) involved in the electrochemical process was calculated to be ~2.0; which indicates that the reaction is a two-electron transfer process.

#### 3.3.2. Chronoamperometry

The catalytic rate constant (*K_cat_*) and diffusion coefficient (D) of catechol at the PEDOT:PSS/20%IL/SPCE sensor were estimated by chronoamperometry. Chronoamperometric measurements were carried out in PBS (pH 7.4) containing various concentrations of catechol (1.0, 2.0, 5.0, 6.0, and 10.0 mM) at an applied potential of +0.5 V (Figure 5B). The catalytic rate constant *K_cat_*, was calculated using the equation [37]:

(4) $$\left(\frac{i_{cat}}{i_{PBS}}\right)=\pi^{½}{\left(K_{cat}.C.t\right)}^{½}$$

where *i_cat_* and *i_BRB_* are the currents obtained at the PEDOT:PSS/20%IL/SPCE sensor for catechol and PBS solution, respectively, *C* is the concentration of catechol, and *t* is time in seconds. The catalytic rate constant was calculated from the slope of the plot of *i_cat_*/*i_PBS_* vs. *t*^1/2^ (insert of Figure 5B) for 1.0 mM catechol concentration. A value of ~6.99 × 10^4^ M^−1^·s^−1^ was calculated for the PEDOT:PSS/20%IL/SPCE sensor, which is satisfactory for the analysis of catechol [33].

The slope of the linear parts of *i* vs. *t*^1/2^ plots (Figure 5C) for the different concentrations of catechol (1.0, 2.0, 5.0, 6.0, and 10 mM) were selected and used to construct the *i·t*^1/2^ vs. C_catechol_ plot (Figure 5D). The slope of *i·t*^1/2^ vs. C_catechol_ plot was used in conjunction with the Cottrel expression [37]:

(5) $$i=\left(\frac{nFAD^{½}C}{\pi^{½}t^{½}}\right)$$

where *i* is current (in A), *n* is the number of electrons (here *n* = 2), *F* is Faraday’s constant, *A* is the electrode area (*A* = 0.12566 cm^2^), *C* is the concentration (1.0 × 10^−6^ mol·cm^−3^), *D* is the diffusion coefficient (cm^2^ s^−1^), and *t* is time (s), to estimate the diffusion coefficient (D) for catechol and was calculated to be ~1.17 × 10^−6^ cm^2^·ps^−1^.

#### 3.3.3. Amperometry in Stirred Solution

**Figure 6:**
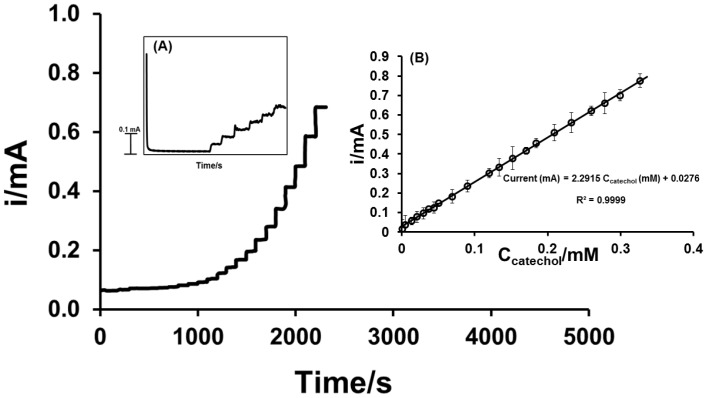
Amperometric responses of the PEDOT:PSS/20%IL/SPCE sensor in stirred PBS (pH 7.4) solution at an applied potential of 0.5 V to varying concentrations of catechol from 0.1 µM to 330.0 µM; insert (**A**) zoom at the first seven standard additions of catechol and (**B**) plot of steady state current vs. catechol concentration.

The amperometric response of catechol in PBS (pH 7.4) was measured on the PEDOT:PSS/20%IL/SPCE sensor at constant potential of 0.5 V, which was the oxidation potential of catechol (a2) (Figure 6). As shown in Figure 6 and the insert 6A, the amperometric current vs. time (*i*-*t*) curve of catechol showed that the PEDOT:PSS/20%IL/SPCE sensor had a rapid response to varying concentrations of catechol in stirred buffer solution. The establishment of well-defined steady-state current responses to standard additions of catechol indicates that the sensor is sensitive. A linear range was recorded from 0.1 µM to 330.0 µM (Figure 6B) with a sensitivity of 18.2 mA·mM·cm^−2^ and a calculated limit of detection (based on 3× the baseline noise) of 23.7 µM; these analytical performance characteristics are considered to be satisfactory for routine analysis of catechol in natural water samples [32,33].

#### 3.3.4. Stability of PEDOT:PSS/20%IL/SPCE Sensor

**Figure 7:**
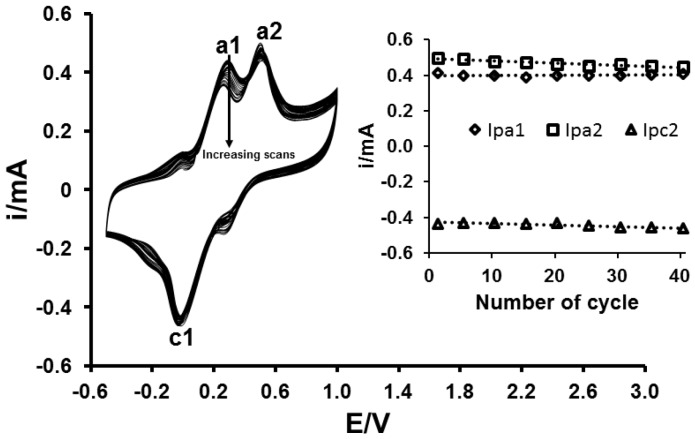
Repetitive cyclic voltammograms (40 scans) recorded at PEDOT:PSS/20%IL/SPCE sensor; insert is peak current vs. cycle number. Voltammograms were recorded in 5.0 mM catechol in PBS (pH 7.4) containing 0.1 M KCl and at a scan rate of 100 mV·s^−1^.

The stability of the conducting polymer composite is crucial for any practical applications. In order to investigate the stability and durability of the electrocatalytic activity of the PEDOT:PSS/20%IL/SPCE sensor, several voltammograms were recorded in catechol solution. In general, unstable electrodes have unstable voltammograms. Figure 7, shows 40 repetitive voltammograms recorded for 5.0 mM catechol and their corresponding anodic (*I_pa_*_1_, *I_pa_*_2_) and cathodic (*I_pc_*) peak currents for selected cycles are shown in Figure 7 (insert). The standard deviation values for *I_pa_*_1_, *I_pa_*_2_, and *I_pc_*_1_ were found to be 1.84%, 0.59%, and 1.27%, respectively. These standard deviation values indicate that the procedure for the sensor fabrication is highly reproducible.

#### 3.3.5. Analysis of Natural Water Samples

To demonstrate the feasibility of the PEDOT:PSS/20%IL/SCPE sensor for routine analysis, the sensor was used to analyze natural water samples. Prior to this analysis, the water samples were analyzed for the presence (or otherwise) of endogenous catechol; this analysis indicated no detectable catechol in the water sampled. After verifying the absence of endogenous catechol in the water samples, amperometry, in conjunction with the method of standard additions [38,39,40,41], was employed to determine the recovery of catechol spiked into the water samples. The analytical performance data for three repeated measurements are summarized in Table 1.

The recoveries were found to be well over 99.0% with coefficient of variations of 0.04 and 0.32. Clearly, the presence of interfering species in the water samples did not have any significant interference with the analysis of the compound; thus, the sensor can be used for routine quantification of catechol in the natural water samples.

## 4. Conclusions

A stable, high-performance composite combining the synergistic effects of the conducting polymer PEDOT:PSS and the room temperature ionic liquid, [EMIM][BF_4_], was formulated and utilized to fabricate a disposable screen-printed sensor. The formulated PEDOT:PSS/IL composite exhibited a highly nano-porous microstructure, excellent stability, and enhanced electrocatalytic properties towards catechol, a priority pollutant. When the sensor was used to analyze catechol, satisfying selectivity and sensitivity data were found. Potential applicability of the sensor in the analysis of catechol in natural water samples was demonstrated with stable, accurate results obtained; which demonstrates that the sensor holds a great promise for routine application in the analysis of this priority pollutant. In the future, sensors based on the transduction capabilities of PEDOT:PSS/IL composite would be developed for biomedical diagnostic applications.

## Figures and Tables

**Table 1:** Recovery of spiked catechol from natural water samples.

Sample	[Catechol]/µM		Mean Recovery (%)
	Amount Added	Amount Found	
**Tap Water**			$\%Recovery=\;\frac{\left(19.94\right)}{20.0}\times 100=99.7$.
***Repeat 1***	20	19.98	
***Repeat 2***	20	19.96	
***Repeat 3***	20	19.89	
**Mean**	-	**19.943**	
**SD**	-	**0.035**	
**CV (%)**	-	**0.177**	
**River Water**			$\%Recovery=\;\frac{\left(19.81\right)}{20.0}\times 100=99.1$
***Repeat 1***	20	19.76	
***Repeat 2***	20	19.88	
***Repeat 3***	20	19.79	
**Mean**	-	**19.81**	
**SD**	-	**0.062**	
**CV (%)**	-	**0.315**	

NB: SD—Standard Deviation; CV—Coefficient of Variation.
